# Role of NAD metabolism-related genes in diabetic nephropathy: subtype classification, biomarker identification, and association with renal function

**DOI:** 10.3389/ebm.2025.10601

**Published:** 2026-01-26

**Authors:** Shengnan Zeng, Yuhong Tao, Hui Guo

**Affiliations:** 1 Department of Pediatric Nephrology, West China Second Hospital, Sichuan University, Chengdu, China; 2 Key Laboratory of Birth Defects and Related Diseases of Women and Children (Sichuan University), Ministry of Education, Chengdu, China

**Keywords:** diabetic nephropathy, redox, immune cell infiltration, bioinformatics, machine learning

## Abstract

Diabetic nephropathy (DN) remains a major complication of diabetes, significantly impacting renal function. Emerging evidence suggests that NAD metabolism plays a crucial role in DN pathogenesis. This study investigates the roles of NAD metabolism-related genes in DN and how there are associated with different disease subtypes. We analyzed gene expression data from DN-associated datasets (GSE30528 and GSE30529) to identify differences in NAD metabolism-related genes between normal and DN samples. We classified DN into subtypes based on NAD gene expression and evaluated NAD scores using ssGSEA. Immune cell infiltration and pathway analyses were assessed using ssGSEA, Microenvironment Cell Populations-counter (MCPcounter), and Gene Set Variation Analysis (GSVA). Key biomarker genes were identified using machine learning algorithms and validated across multiple datasets. We further explored the relationship between gene expression and kidney function using the Nephroseq V5 tool. Thirteen differentially expressed NAD metabolism-related genes were identified, with distinctive expression patterns observed between normal and DN samples. Two distinct NAD-related subtypes were classified, demonstrating significant differences in gene expression, immune cell infiltration, and pathway activities. Immune-related pathways and cellular processes exhibited varied enrichment between subtypes. Six key NAD metabolism-related genes (FMO3, ALDH1A3, FMO5, TKT, LBR, HPGD) were identified as potential biomarkers. Higher levels of FMO3, ALDH1A3, TKT, and LBR were linked to worse kidney function, while FMO5 and HPGD were associated with better kidney function. The study highlights the significant involvement of NAD metabolism-related genes in DN pathogenesis and their association with disease subtypes and renal function. The identified biomarkers could be targets for new treatments and provide insight for future DN research.

## Impact statement

Our findings highlight the significant involvement of NAD metabolism-related genes in DN pathogenesis and underscore their potential as biomarkers for disease classification and therapeutic intervention. This study provides a foundation for future research into DN mechanisms and underscores the translational potential of targeting NAD metabolism in DN treatment strategies.

## Introduction

Diabetic nephropathy (DN) is acknowledged as a primary contributor to end-stage renal disease (ESRD) and a serious complication of diabetes mellitus, impacting millions globally [1]. The progressive characteristics of DN, which include albuminuria, a diminishing glomerular filtration rate (GFR), and eventual renal failure, place a significant strain on healthcare systems and highlight the urgent need for effective diagnostic and treatment approaches [2, 3]. The development of DN is influenced by multiple factors and involves a complex interaction of hemodynamic and metabolic elements. Persistent hyperglycemia triggers various pathogenic mechanisms, such as the activation of the renin-angiotensin-aldosterone system (RAAS), increased formation of advanced glycation end products (AGEs), oxidative stress, inflammation, and fibrosis [4–6]. These interconnected processes lead to glomerular hypertrophy, thickening of the basement membrane, loss of podocytes, and tubulointerstitial fibrosis, ultimately resulting in progressive renal impairment and ESRD [7, 8].

Nicotinamide adenine dinucleotide (NAD) metabolism plays a pivotal role in cellular bioenergetics, redox reactions, and signaling pathways [9]. NAD serves as a coenzyme in redox reactions, crucial for ATP production and cellular metabolism [10]. It also functions as a substrate for enzymes involved in post-translational modifications, such as sirtuins and poly (ADP-ribose) polymerases (PARPs), which are essential for DNA repair, gene expression regulation, and maintaining genomic stability [11, 12]. In the context of chronic kidney disease and DN, NAD metabolism has garnered attention due to its involvement in inflammatory and oxidative stress responses, mitochondrial dysfunction, and cellular senescence [13, 14]. Studies have demonstrated that NAD levels decline with age and in various disease states, including CKD and DN, contributing to exacerbated renal damage and impaired renal function [15–17]. NAD+ supplementation has been shown to ameliorate kidney injury in animal models, highlighting its potential as a therapeutic target [18].

**FIGURE 1:**
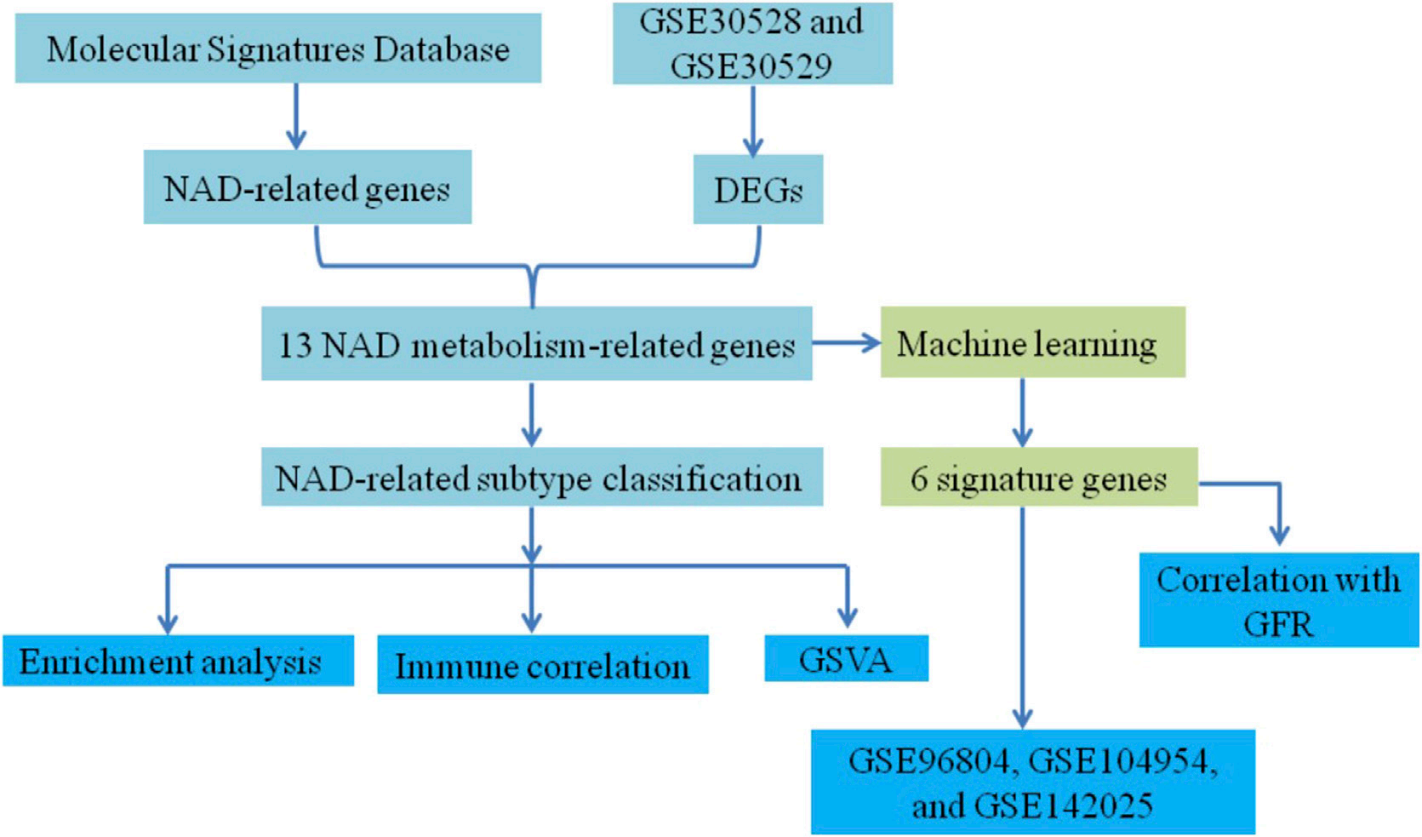
Flowchart of the study.

However, despite these findings, the specific roles of NAD metabolism-related genes in DN pathogenesis and their potential as biomarkers for disease subtypes and renal function remain poorly understood. This study aims to fill this knowledge gap by investigating the expression and function of NAD metabolism-related genes in DN. We will classify DN subtypes based on their expression profiles and identify potential biomarkers that correlate with renal function. Figure 1 presents the flow chart of the study. Initially, NAD-related genes were extracted from the Molecular Signatures Database and differentially expressed genes (DEGs) were identified from datasets GSE30528 and GSE30529. Combining these sources, 13 NAD metabolism-related genes were determined. This set of genes underwent machine learning analysis, resulting in the identification of 6 signature genes. Concurrently, these 13 genes were used to classify samples into NAD-related subtypes, followed by enrichment analysis, immune correlation studies, and Gene Set Variation Analysis (GSVA). Finally, the expression of six marker genes was validated using additional datasets GSE96804, GSE104954, and GSE142025. The correlation between these marker genes and glomerular filtration rate (GFR) was assessed via the Nephroseq V5 tool, comprehensively elucidating their underlying mechanisms in DN.

Our findings highlight the significant involvement of NAD metabolism-related genes in DN pathogenesis and underscore their potential as biomarkers for disease classification and therapeutic intervention. This study provides a foundation for future research into DN mechanisms and underscores the translational potential of targeting NAD metabolism in DN treatment strategies.

## Materials and methods

### Data collection and preprocessing

The gene expression datasets GSE30528 and GSE30529 were obtained from the Gene Expression Omnibus (GEO) database. The raw expression matrix was extracted using the Affy package (version 1.86.0). Gene annotations were mapped using the GPL571 platform annotation file. For genes represented by multiple probes, the expression value was calculated as the average expression of the corresponding probes. Genes and samples with more than 50% missing values were excluded from further analysis to maintain data integrity. Finally, to standardize gene expression levels across samples, median normalization was applied.

### Identification of NAD metabolism-related genes

Differential expression analysis of NAD metabolism-related genes between DN and normal samples was performed using the limma package (version 3.52.2) in R software (version 4.2.1). The analysis employed empirical Bayes statistics with moderated t-tests to identify significantly differentially expressed genes. Statistical significance was determined using the adjusted p-value < 0.05 (false discovery rate correction using the Benjamini-Hochberg method). Genes meeting the threshold were considered significantly differentially expressed and included in subsequent analyses. A volcano plot was generated to visualize the DEGs, and a heatmap was created using the Complexheatmap package (version 2.13.1) to illustrate the expression patterns of the identified NAD metabolism-related genes. Box plots were constructed to further delineate the expression levels, confirming statistically significant differences. Correlation analysis among the NAD metabolism-related genes was conducted using the ggplot2 package (version 3.4.4), and results were visualized in a correlation pie chart.

### NAD score evaluation

The single-sample Gene Set Enrichment Analysis (ssGSEA) algorithm from the GSVA package (version 1.44.5) was utilized to calculate NAD scores for each sample. The NAD metabolism gene sets were obtained from Molecular Signatures Database, including six pathways: GOBP_CELL_REDOX_HOMEOSTASIS, GOBP_NADP_METABOLIC_PROCESS, GOMF_NAD_BINDING, GOMF_NADP_BINDING, GOMF_NADPH_BINDING, and GOBP_NADPH_REGENERATION. The expression profiles of the genes within these sets were used to compute the enrichment scores for each sample. Normalization was performed using the z-score transformation to ensure comparability across samples.

### Subtype classification

Based on the expression profiles of the 13 NAD metabolism-related genes, samples were classified into two NAD-related subtypes (subtype1 and subtype2) utilized the ConsensusClusterPlus package. The consensus clustering was performed with the following parameters: Pearson correlation distance metric, partitioning around medoids (PAM) clustering algorithm, maximum cluster number (k) tested from 2 to 10, 10 resampling iterations, and 80% subsampling ratio. The optimal number of clusters was determined based on the consensus matrix heatmap, consensus cumulative distribution function (CDF), and delta area plot. Cluster stability was assessed using silhouette analysis, and the clustering solution with the highest average silhouette score and most stable consensus was selected. Differential expression analysis between these subtypes was performed, and a volcano plot was generated to visualize the results.

### Enrichment analysis

Gene Ontology (GO) and Kyoto Encyclopedia of Genes and Genomes (KEGG) pathway enrichment analyses were conducted using the clusterProfiler package (version 4.4.4). The results were visualized using lollipop and bubble plots to identify significant biological processes and pathways associated with the DEGs.

### Immune cell infiltration analysis

The ssGSEA algorithm was again applied to assess immune cell infiltration levels between the two NAD-related subtypes. The MCPcounter package (version 1.2.0) was utilized to further evaluate immune cell types, and correlation analyses were performed to explore associations between NAD scores and immune cell infiltration levels.

### Machine learning for biomarker identification

To identify key NAD metabolism-related biomarker genes, three machine learning algorithms were employed: LASSO regression, Random Forest (RF), and Support Vector Machine-Recursive Feature Elimination (SVM-RFE). For LASSO regression, the regularization parameter (lambda) was optimized through 10-fold cross-validation using cv.glmnet function, selecting the lambda value that minimized cross-validation error (lambda.min). For Random Forest, we used the randomForest package (version 4.7.1.1), setting the number of trees (ntree) to 500 and the number of variables tried at each split (mtry) to the square root of the total number of features. Hyperparameter tuning was conducted using grid search combined with 10-fold cross-validation to find the best mtry value. For SVM-RFE, the e1071 package (version 1.7.13) was used with a linear kernel. Feature selection was performed recursively, and the optimal number of features was determined through 10-fold cross-validation. The optimal features were determined, and a Venn diagram was created to illustrate the common genes identified across the algorithms.

### Validation of biomarker genes

The expression of key NAD metabolism-related marker genes was validated using three independent datasets (GSE96804, GSE104954, and GSE142025). The relevant information for those datasets is presented in Supplementary Table S1.

### Association with renal function

The relationship between NAD metabolism-related hub genes and renal function in DN patients was evaluated using the Nephroseq V5 tool.^1^ The glomerular filtration rate (GFR) was used as a measure of renal function. Correlation analyses were performed to assess the associations between the expression levels of NAD metabolism-related hub genes and GFR. These analyses were conducted to provide a comprehensive understanding of the impact of gene expression on renal function in DN patients.

## Results

### Analysis of differentially expressed NAD metabolism-related genes in DN

**FIGURE 2:**
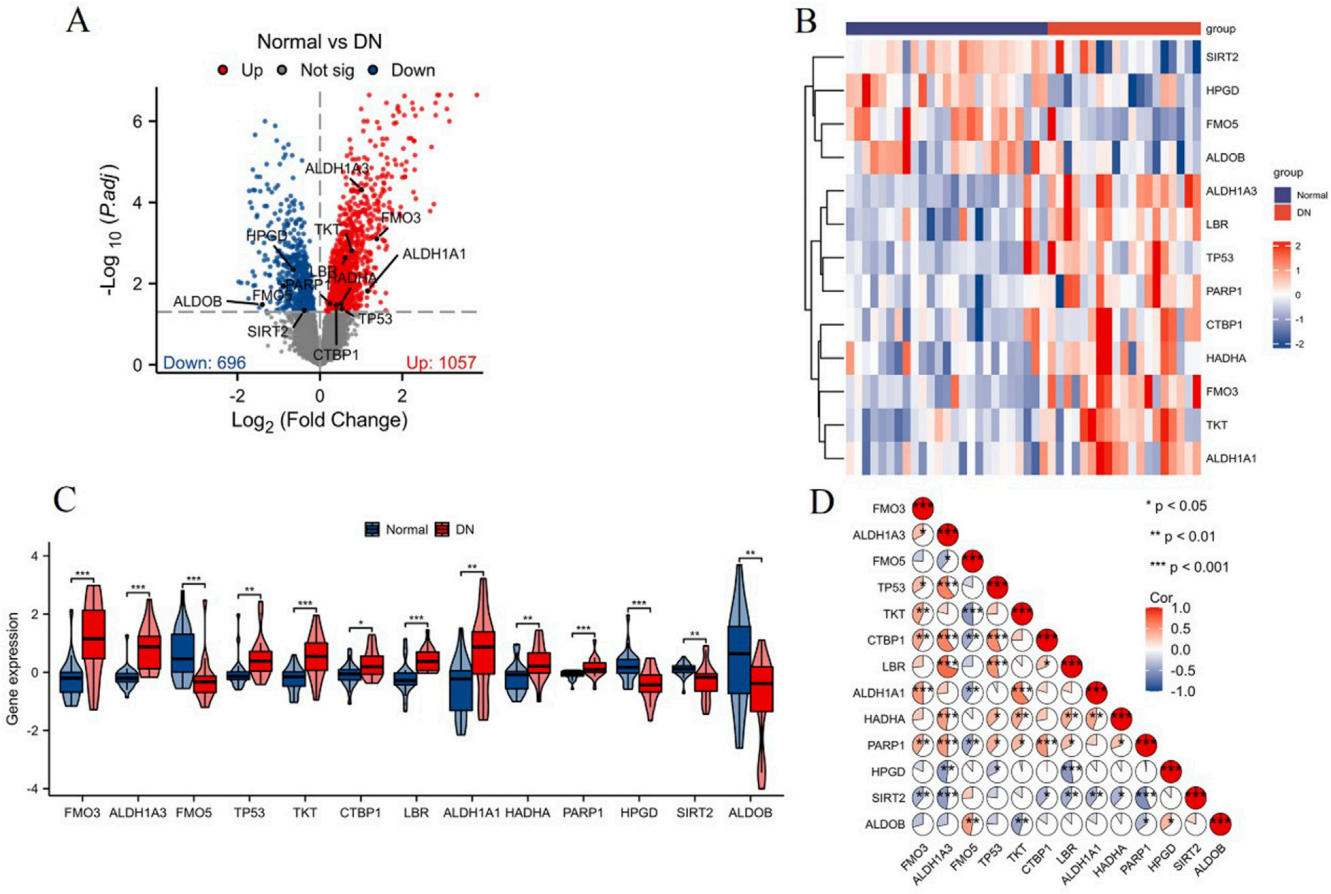
Analysis of differentially expressed NAD metabolism-related genes in DN. **(A)** Volcano plot showing 13 differentially expressed NAD metabolism-related genes identified in the combined datasets (GSE30528 and GSE30529). Significantly upregulated genes are highlighted in red, while downregulated genes are highlighted in blue. **(B)** Heatmap illustrating the expression levels of these 13 genes across normal and DN samples. Red indicates higher expression, and blue indicates lower expression. **(C)** Box plots showing the expression distribution of the 13 NAD metabolism-related genes in normal (blue) and DN (red) samples. Statistically significant differences are indicated by *p < 0.05, **p < 0.01, ***p < 0.001. **(D)** Correlation pie chart displaying the correlations among the 13 NAD metabolism-related genes. Positive correlations are shown in red, negative correlations in blue, with the significance levels noted as *p < 0.05, **p < 0.01, ***p < 0.001.

NAD metabolism is essential for many cellular processes, including energy production, DNA repair, and regulation of cellular stress responses. Given its central role in maintaining cellular homeostasis, dysregulation of NAD metabolism is implicated in various diseases, including diabetic nephropathy (DN). Therefore, identifying differentially expressed NAD metabolism-related genes in DN could provide insights into the molecular mechanisms underlying this disease. The analysis of differential expression in the DN-associated combined datasets (GSE30528 and GSE30529) identified 13 differentially expressed NAD metabolism-related genes, visualized in the volcano plot (Figure 2A). Expression profiling of these 13 genes was conducted and illustrated through a heatmap, showcasing distinct expression patterns between normal and DN samples (Figure 2B). The corresponding box plot further delineates the expression levels of these genes, with statistically significant differences observed (**p < 0.01 and ***p < 0.001), reinforcing the differential expression identified (Figure 2C). Correlation analysis of the 13 NAD metabolism-related genes was visualized using a correlation pie chart (Figure 2D). This analysis underscored significant correlations among the genes, suggesting intricate co-regulatory mechanisms within the context of NAD metabolism in DN. Moreover, the NAD metabolism-related genes played a role in pathways related to fatty acid metabolism (Supplementary Figure S1). Overall, these findings highlight the critical role of NAD metabolism-related genes in DN, laying the groundwork for future research into their functional roles and potential as therapeutic targets.

### NAD score evaluation and subtype classification in DN

**FIGURE 3:**
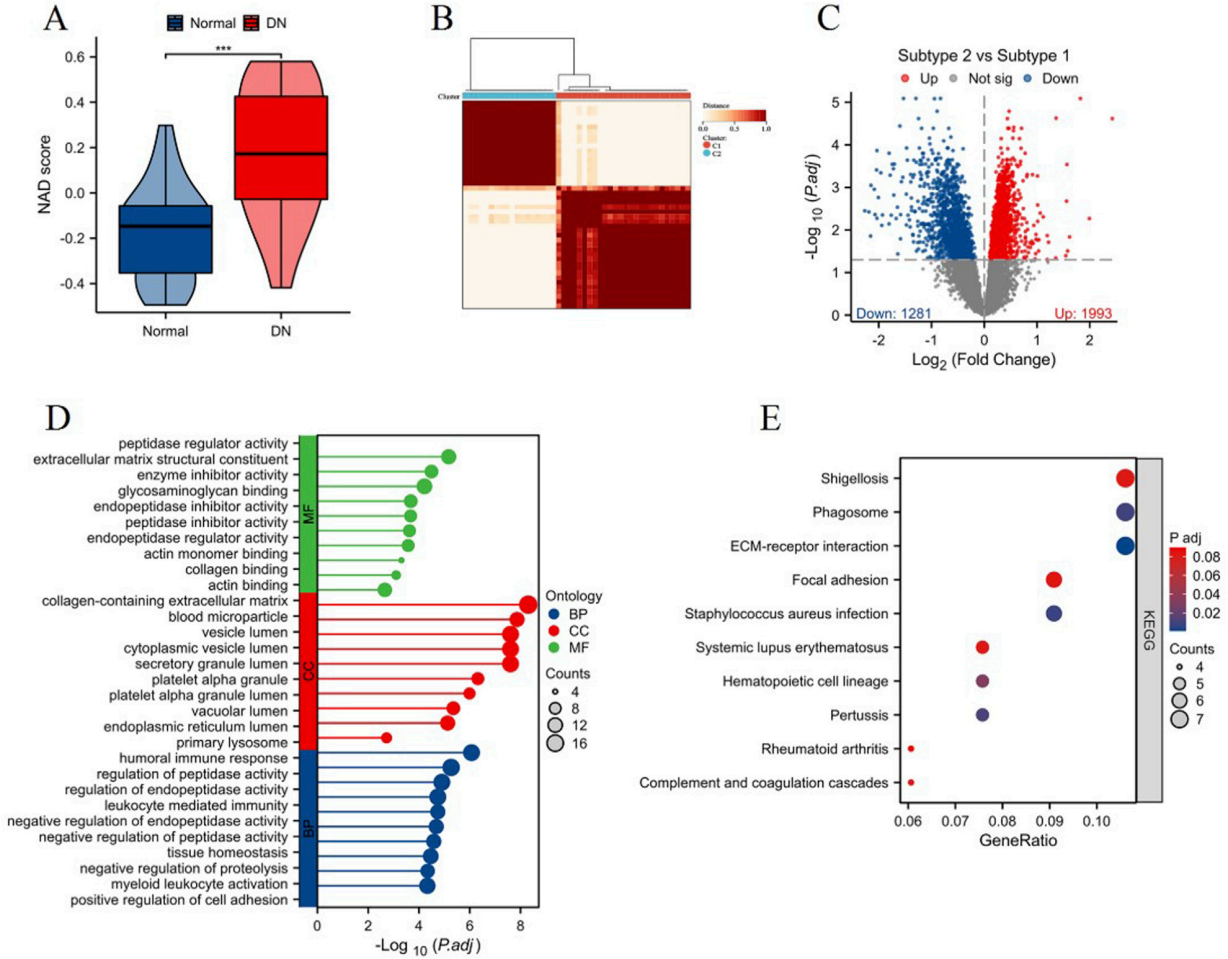
Analysis of NAD score and NAD-related subtypes in DN. **(A)** Violin plot showing the NAD score levels in control (blue) and DN (red) groups, assessed using the ssGSEA algorithm. **(B)** Heatmap depicting the clustering of samples into two NAD-related subtypes (C1 and C2) based on the expression profiles of 13 differentially expressed NAD-related genes. **(C)** Volcano plot displaying the differential expression analysis between subtypes C1 and C2. Significant upregulated genes in C2 compared to C1 are shown in red, and downregulated genes are shown in blue. **(D)** Gene Ontology (GO) enrichment analysis of differentially expressed genes between C1 and C2 subtypes, showing significantly enriched molecular functions (MF), cellular components (CC), and biological processes (BP). **(E)** KEGG pathway enrichment analysis illustrating the significant pathways associated with differentially expressed genes between the two subtypes.

Using the ssGSEA algorithm, we assessed the NAD score levels between the control group and the DN group. The NAD score was significantly increased in the DN group compared to the control group, as illustrated in Figure 3A (***p < 0.001). Based on the expression profiles of the 13 differentially expressed NAD-related genes, we classified the samples into two distinct NAD-related subtypes: C1 and C2 (Figure 3B). Differential expression analysis between the C1 and C2 subtypes is presented in Figure 3C. The volcano plot depicts a significant number of genes being differentially expressed between the subtypes, with 1993 genes upregulated and 1281 genes downregulated in subtype C2 compared to subtype C1. Enrichment analysis of DEGs between C1 and C2 highlighted several key biological processes and signaling pathways. The Gene Ontology (GO) enrichment analysis (Figure 3D) revealed significant enrichment in molecular functions such as peptidase regulator activity and collagen binding, cellular components including extracellular matrix and secretory granule lumen, and biological processes such as humoral immune response, leukocyte mediated immunity, regulation of endopeptidase activity and cell adhesion. The KEGG pathway enrichment analysis (Figure 3E) identified several pathways significantly associated with the DEGs, including ECM-receptor interaction, focal adhesion, and phagosome. Notably, pathways involved in immune response and infection, such as systemic lupus erythematosus and *Staphylococcus aureus* infection, were also enriched, indicating potential roles in DN pathogenesis. The results of the functional enrichment analysis are presented in Supplementary Table S2. These results collectively highlight the heterogeneity in NAD metabolism-related gene expression in DN and underscore the relevance of specific pathways and biological processes in the disease pathology, providing valuable insights into potential therapeutic targets for DN.

### Immune cell infiltration analysis in NAD-related subtypes of DN

**FIGURE 4:**
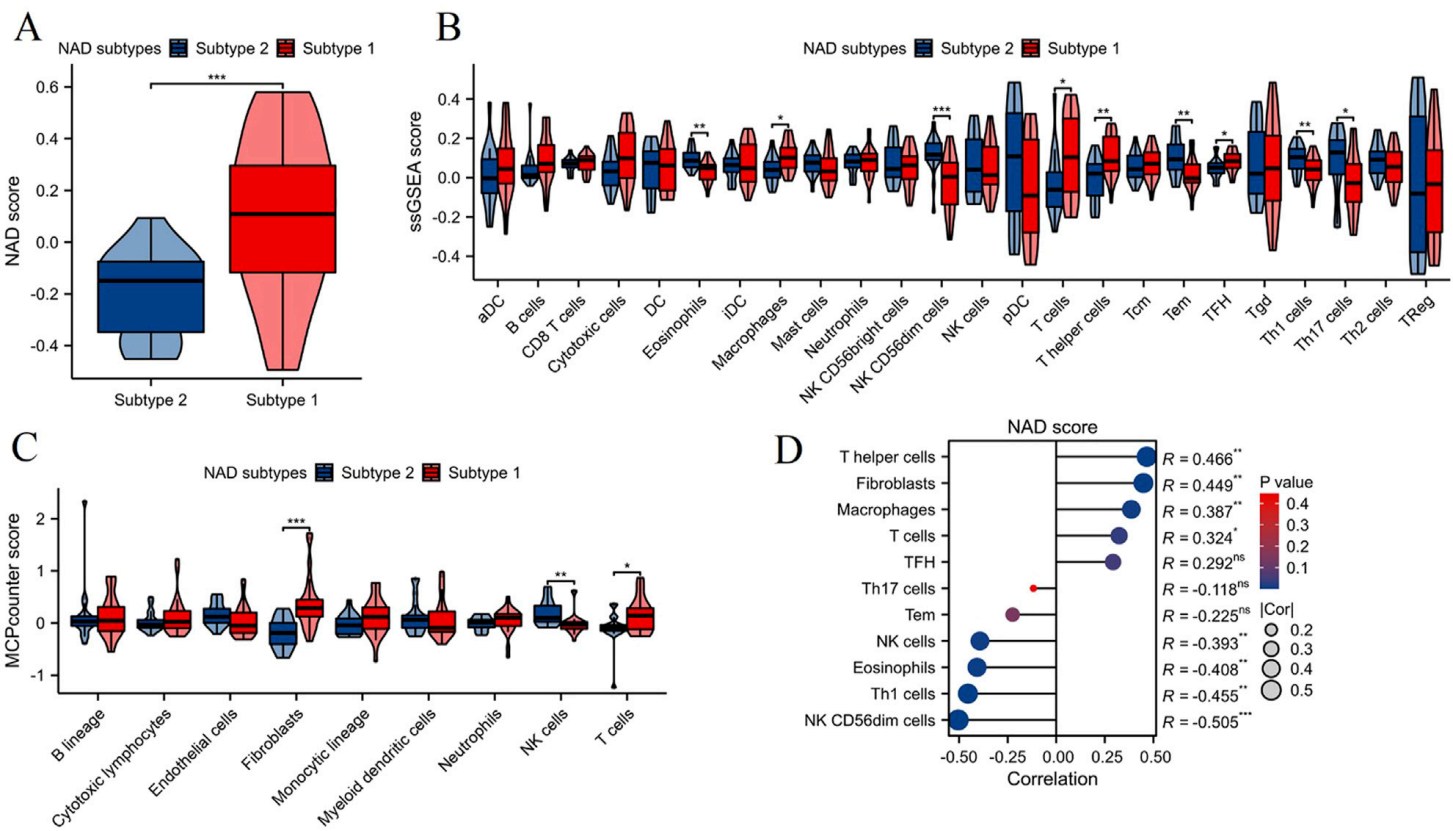
NAD score and immune cell infiltration analysis in NAD-related subtypes of DN. **(A)** Violin plot showing the NAD score levels in the two NAD-related subtypes (Subtype 1 and Subtype 2). **(B)** Immune cell infiltration levels assessed by the ssGSEA algorithm between the two subtypes. Significant differences in several immune cell types are marked (*p < 0.05, **p < 0.01, ***p < 0.001). **(C)** Immune cell infiltration levels assessed by the MCPcounter algorithm between the two subtypes. Significant differences in infiltration levels are indicated (*p < 0.05, **p < 0.01, ***p < 0.001). **(D)** Correlation analysis between NAD scores and immune cell infiltration levels.

Using the ssGSEA algorithm, we assessed the NAD score levels between the two NAD-related subtypes (Subtype 1 and Subtype 2). The results, depicted in Figure 4A, show that Subtype 1 exhibited a significantly higher NAD score compared to Subtype 2 (***p < 0.001). This indicates a differential NAD metabolic state between the two subtypes. We then evaluated the levels of immune cell infiltration between the two subtypes using the ssGSEA algorithm. As shown in Figure 4B, Subtype 1 had significantly higher infiltration levels of various immune cells including macrophages, T cells, TFH, and T helper cells. Complementing the ssGSEA analysis, the MCPcounter algorithm was utilized to further assess the immune cell infiltration between the two subtypes (Figure 4C). Consistent with the ssGSEA results, Subtype 1 showed significantly higher levels of fibroblasts and T cells compared to Subtype 2. These findings reinforce the notion of varied immune profiles between the NAD-related subtypes. Correlation analysis between NAD scores and immune cell infiltration levels revealed significant associations (Figure 4D). Specifically, NAD scores positively correlated with the infiltration levels of T helper cells, fibroblasts, and macrophages. Conversely, negative correlations were observed with NK CD56dim cells, Th1 cells, eosinophils, and NK cells. These results collectively demonstrate that NAD metabolic states are closely linked with immune cell infiltration profiles, offering valuable insights into the pathophysiological mechanisms underlying DN and potential avenues for targeted therapies.

### Immune-related pathway analysis and correlation with NAD score in NAD-related subtypes of DN

**FIGURE 5:**
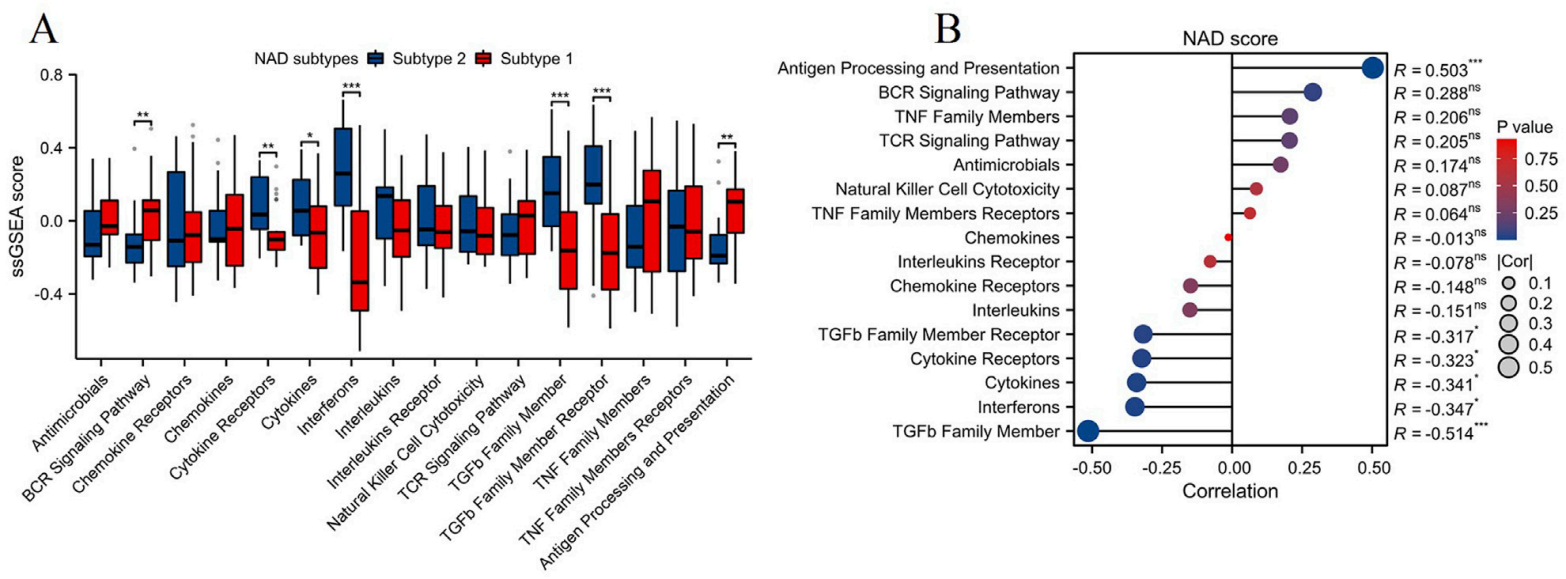
Immune-related pathway analysis and its correlation with NAD score in NAD-related subtypes of DN. **(A)** Box plot showing the levels of immune-related pathways in the two NAD-related subtypes (Subtype 1 and Subtype 2) assessed by the ssGSEA algorithm. Significant differences between the subtypes in various pathways are indicated (*p < 0.05, **p < 0.01, ***p < 0.001). **(B)** Correlation analysis between NAD scores and the levels of immune-related pathways.

The ssGSEA algorithm was employed to evaluate the levels of immune-related pathways between the two NAD-related subtypes (Subtype 1 and Subtype 2). As illustrated in Figure 5A, Subtype 1 exhibited significantly higher activity in several immune-related pathways compared to Subtype 2. Notably, pathways such as BCR signaling pathway and antigen processing and presentation were significantly upregulated in Subtype 1 (**p < 0.01). Correlation analysis between NAD scores and immune-related pathways revealed significant associations, as shown in Figure 5B. The most prominent positive correlation was observed between the NAD score and the antigen processing and presentation pathway (R = 0.503, ***p < 0.001). In contrast, negative correlations were noted between the NAD score and pathways such as the TGFβ family member, interferons, and cytokines (ranging from R = −0.341 to R = −0.514, *p < 0.05, ***p < 0.001). These observations indicate that higher NAD scores are associated with increased involvement in antigen processing and presentation, while decreasing activity in other immune-modulatory pathways. These findings collectively suggest that NAD metabolism variations affect immune pathway activities differentially across the NAD-related subtypes in DN. This could provide a foundation for understanding the mechanistic links between NAD metabolism and immune responses, offering potential therapeutic targets for managing diabetic kidney disease through modulating NAD-associated immune pathways.

### GSVA enrichment analysis and correlation with NAD score in NAD-related subtypes

**FIGURE 6:**
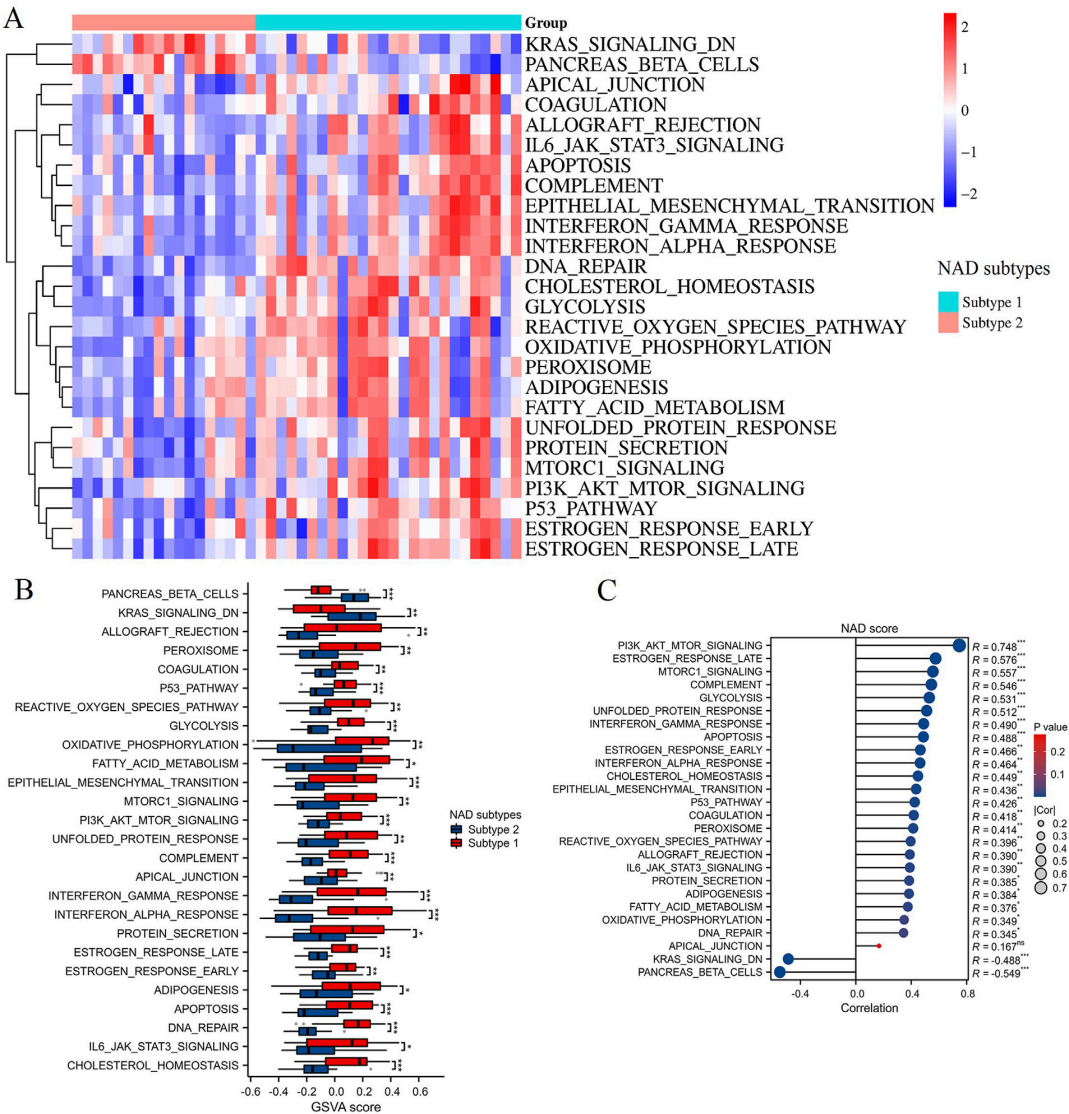
GSVA enrichment analysis. **(A)** Heatmap displaying the GSVA enrichment scores for various pathways between Subtype 1 and Subtype 2. Red indicates high enrichment, and blue indicates low enrichment. **(B)** Box plots showing the GSVA scores for significantly different pathways between NAD-related subtypes. Subtype 1 is represented in red, and Subtype 2 is represented in blue. **(C)** Correlation analysis between NAD scores and pathways differentially enriched in the NAD-related subtypes. *p < 0.05, **p < 0.01, ***p < 0.001.

The GSVA enrichment analysis was conducted to evaluate pathway-level differences between the two NAD-related subtypes (Subtype 1 and Subtype 2). The heatmap in Figure 6A illustrates significant variations in pathway activity across the subtypes. Enriched pathways in Subtype 2 include KRAS signaling and pancreas beta cells, while Subtype 1 shows enrichment in pathways such as apoptosis, peroxisome, glycolysis, interferon gamma response, fatty acid metabolism, etc. These pathways are functionally relevant, as KRAS signaling is critical for cell proliferation and survival, while pancreatic beta cells are essential for insulin secretion and glucose homeostasis. The box plots in Figure 6B provide a more detailed comparison of the GSVA scores between the subtypes. Notably, pathways like KRAS signaling and pancreatic beta-cells functionality are significantly more enriched in Subtype 2 compared to Subtype 1. Conversely, Subtype 1 exhibits higher enrichment in pathways such as the reactive oxygen species pathway, fatty acid metabolism, p53 pathway, apoptosis, PI3K/AKT/mTOR signaling, glycolysis, interferon alpha response, and interferon gamma response. These pathways are integral to cellular stress responses, energy metabolism, and immune regulation, suggesting that Subtype 1 may be more responsive to metabolic dysregulation and oxidative stress. Correlation analysis between the NAD score and the differentially enriched pathways is presented in Figure 6C. Strong positive correlations were observed between the NAD score and pathways such as PI3K/AKT/mTOR signaling, estrogen response late, and MTORC1 signaling (R = 0.748, R = 0.576, R = 0.557; ***p < 0.001). Negative correlations were noted for pathways like pancreatic beta-cells and KRAS signaling DN (R = −0.549, R = −0.488; ***p < 0.001). These findings highlight the heterogeneity in pathway activities between the two NAD-related subtypes and their association with NAD metabolism. This underscores the importance of specific signaling and metabolic pathways in the context of DN, providing potential targets for therapeutic intervention.

### Identification of key NAD metabolism-related biomarker genes in DN using machine learning algorithms

**FIGURE 7:**
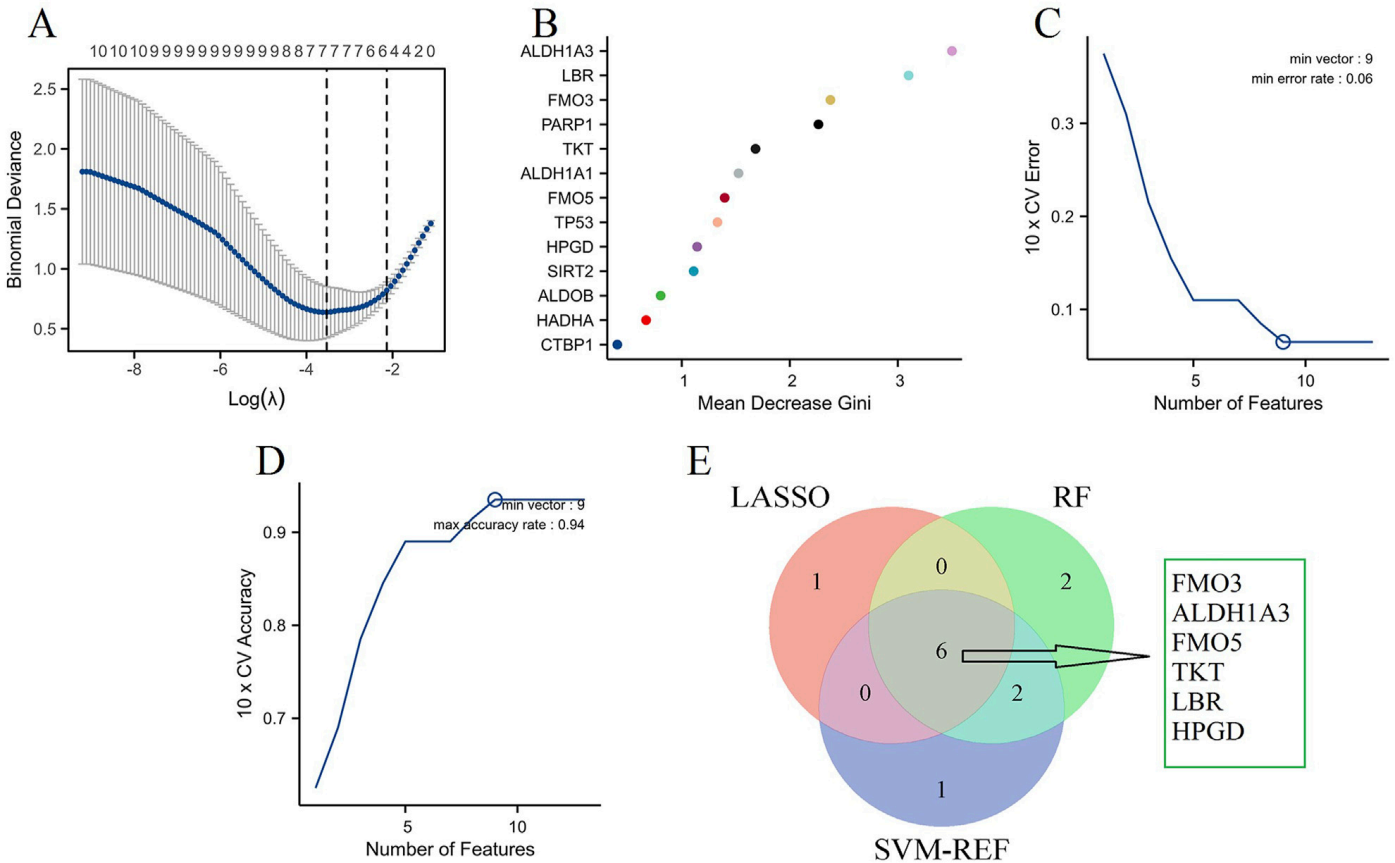
Machine learning algorithms. **(A)** LASSO regression analysis identifying significant genes based on the optimal λ value, as determined by binomial deviance. **(B)** RF analysis showing the mean decrease in Gini index for each gene, indicating their importance in distinguishing DN. **(C)** SVM-RFE analysis identifying the optimal number of features based on the 10-fold cross-validation (CV) error rate. **(D)** SVM-RFE analysis depicting the 10-fold CV accuracy for various numbers of features. **(E)** Venn diagram integrating results from LASSO, Random Forest, and SVM-RFE.

To identify key NAD metabolism-related biomarker genes in DN patients, we utilized three machine learning algorithms: LASSO, Random Forest (RF), and Support Vector Machine-Recursive Feature Elimination (SVM-RFE). The LASSO regression model was applied to select 7 critical features, and the binomial deviance indicated an optimal λ value (Figure 7A). Next, the RF algorithm was employed to evaluate feature importance. As shown in Figure 7B, the mean decrease in Gini index was calculated for each gene, identifying 10 top-performing genes critical for distinguishing between DN subtypes. Similarly, the SVM-RFE approach was applied to determine the optimal number of features by evaluating the cross-validation (CV) error rate (Figure 7C) and the associated CV accuracy (Figure 7D). The analysis suggested that a minimal error rate and maximal accuracy were achieved with nine features, confirming the robustness of these selected genes. Integrating the results from all three machine learning methods, the Venn diagram in Figure 7E illustrates the common genes identified by LASSO, RF, and SVM-RFE. Six key NAD metabolism-related genes (FMO3, ALDH1A3, FMO5, TKT, LBR, and HPGD) were consistently selected across the three algorithms, indicating their potential as reliable biomarkers for DN.

### Validation of key NAD metabolism-related marker genes in DN

**FIGURE 8:**
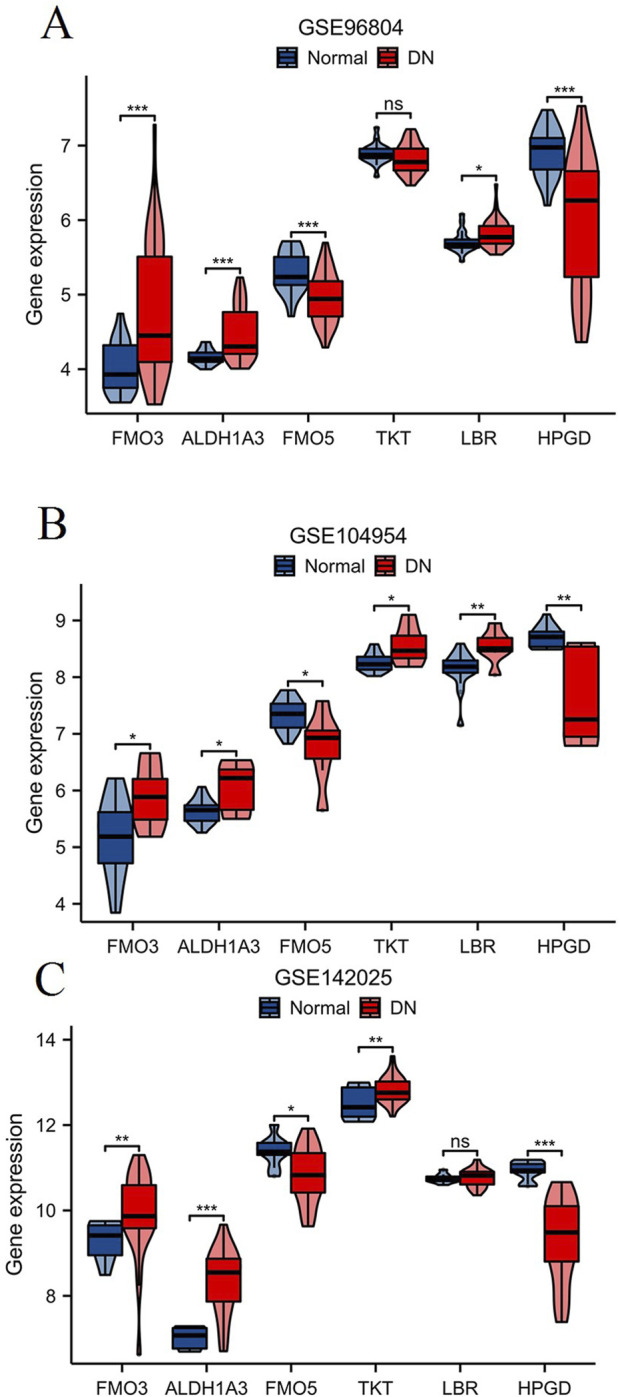
Validation of key NAD metabolism-related genes in DN. The expression levels of six NAD metabolism-related genes (FMO3, ALDH1A3, FMO5, TKT, LBR, HPGD) were analyzed in three independent DN-associated datasets: GSE96804 **(A)**, GSE104954 **(B)**, and GSE142025 **(C)**. *p < 0.05, **p < 0.01, ***p < 0.001.

To validate the expression of key NAD metabolism-related marker genes in DN, we analyzed three independent DN-associated datasets: GSE96804 (Figure 8A), GSE104954 (Figure 8B), and GSE142025 (Figure 8C). In all three datasets, FMO3, ALDH1A3, FMO5, and HPGD exhibited consistent and significant dysregulation in DN samples. Furthermore, analysis of the GSE104954 and GSE142025 datasets revealed a marked upregulation of TKT expression in DN samples, while LBR expression was notably increased in GSE96804 and GSE104954. These results suggest that FMO3, ALDH1A3, FMO5, and HPGD are potential key marker genes in the context of NAD metabolism associated with DN.

### Evaluation of NAD metabolism-related hub genes’ association with renal function in DN patients

**FIGURE 9:**
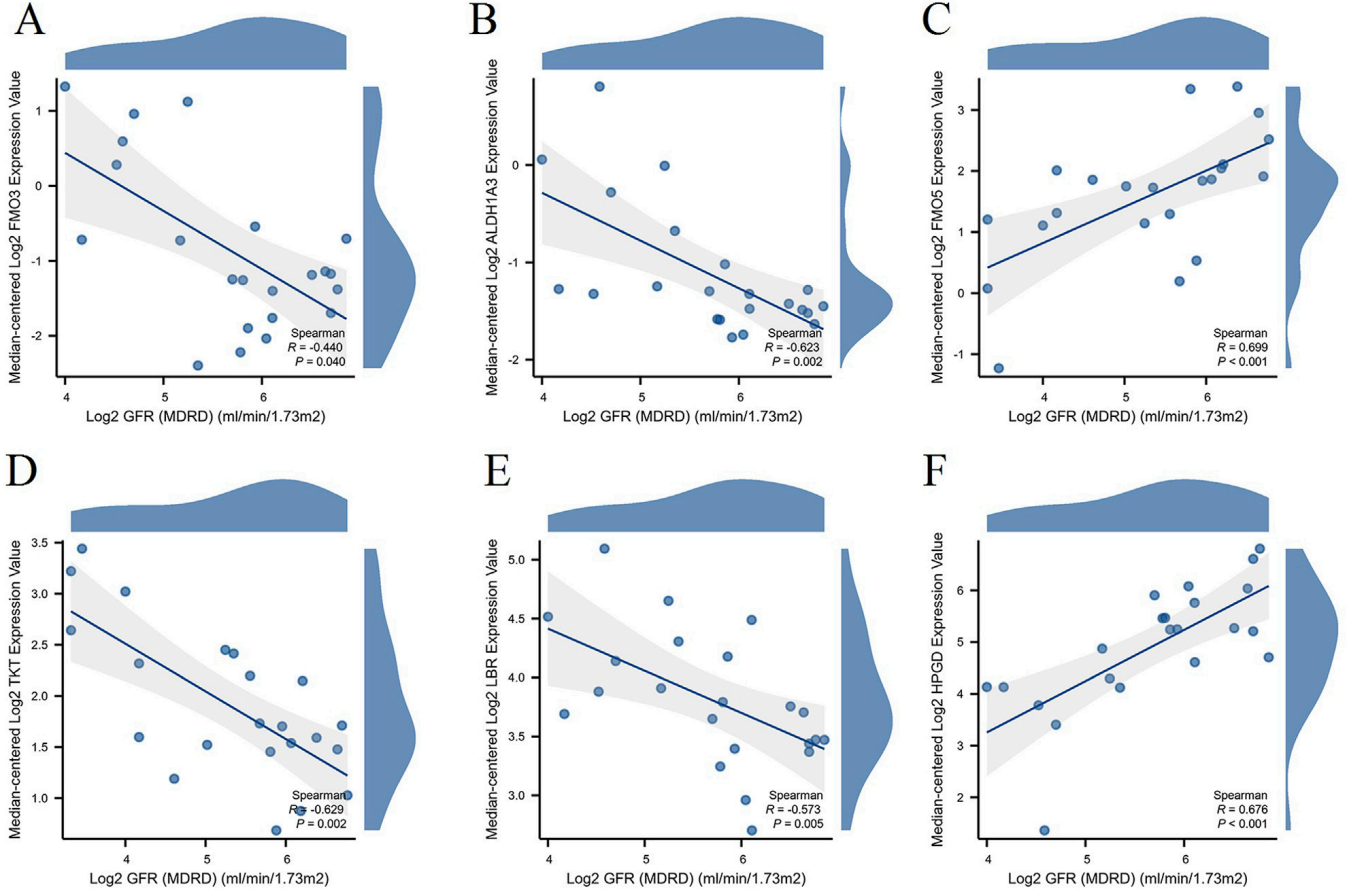
Correlation between hub gene expression and renal function in DN Patients. Scatter plots illustrating the correlation between the expression levels of hub genes and renal function, represented by Log2 GFR (MDRD), in DN patients. The genes analyzed include FMO3 **(A)**, ALDH1A3 **(B)**, FMO5 **(C)**, TKT **(D)**, LBR **(E)**, and HPGD **(F)**.

Using the Nephroseq V5 tool,^1^ we evaluated the relationship between key NAD metabolism-related hub genes and renal function in DN patients. The renal function was assessed via the estimated glomerular filtration rate (GFR) (Figure 9). The expression levels of NAD metabolism-related hub genes show significant correlations with renal function in DN patients. Specifically, FMO3 (R = −0.44, p = 0.04), ALDH1A3 (R = −0.623, p = 0.002), TKT (R = −0.629, p = 0.002), and LBR (R = −0.573, p = 0.005) are inversely correlated with GFR, suggesting their increased expression is associated with worsening renal function. In contrast, FMO5 (R = 0.699, p < 0.001) and HPGD (R = 0.676, p < 0.001) are positively correlated with GFR, indicating their increased expression is linked with better renal function. These findings underscore the potential roles of these genes in the regulation of renal function in DN and their utility as biomarkers.

## Discussion

DN is a significant complication of diabetes, leading to increased morbidity and mortality among affected individuals. Recent studies have highlighted the critical role of NAD metabolism in various metabolic disorders, including DN. Our study identified thirteen differentially expressed NAD metabolism-related genes in DN, with a notable increase in the NAD score among DN patients compared to controls. We classified two distinct NAD-related subtypes, revealing significant differences in gene expression, immune cell infiltration, and pathway activities. Key biomarkers, including FMO3, ALDH1A3, FMO5, TKT, LBR, and HPGD, were identified, with varying correlations to renal function.

Our findings align with previous research indicating that NAD metabolism is integral to the pathogenesis of DN. For instance, the altered NAD levels can influence oxidative stress and inflammation, both of which are critical in DN progression [19–21]. Additionally, NAD+ precursor administration mitigates inflammatory responses in the context of renal injury, suggesting a protective role of NAD metabolism in DN [22]. The identification of specific NAD-related subtypes in our study adds a new dimension to existing literature, suggesting that personalized approaches based on NAD metabolism could enhance therapeutic strategies. Moreover, research reinforces our conclusions by demonstrating that NAD metabolism can enhance mitochondrial function and decrease inflammation in DN, thereby underscoring the potential for therapeutic strategies that focus on NAD metabolism [20].

The identified biomarkers demonstrate distinct biological roles in DN pathogenesis through multiple mechanisms. FMO3 (Flavin-containing monooxygenase 3) plays a crucial role in xenobiotic metabolism and has been implicated in renal protection mechanisms. Beyond our finding that FMO3 deficiency confers renal protection following ischemia-reperfusion injury in murine models [23], recent studies demonstrate that FMO3 modulates trimethylamine N-oxide (TMAO) production, which is elevated in diabetic patients and correlates with renal dysfunction [24]. ALDH1A3 (Aldehyde dehydrogenase 1A3) serves as a critical enzyme in aldehyde detoxification and retinoic acid synthesis. Inhibition of ALDH1A3, whether through genetic means or pharmacological intervention, has been shown to reduce blood glucose levels and enhance insulin secretion in diabetic mice [25]. FMO5 is crucial in regulating diverse metabolic pathways and processes, notably those associated with lipid homeostasis and the absorption and metabolism of glucose [26]. It serves as a key regulator of body weight, glucose disposal, and insulin sensitivity [27]. TKT (Transketolase) is essential for the pentose phosphate pathway and NADPH generation [28]. TKT deficiency leads to a reduction in thioredoxin-interacting protein levels, which is a recognized inhibitor of GLUT4. This occurs by diminishing NADPH and glutathione levels, subsequently inducing oxidative stress in brown adipose tissue [29]. LBR (Lamin B receptor) is involved in nuclear envelope integrity and has emerging roles in metabolic regulation [30]. HPGD (15-hydroxyprostaglandin dehydrogenase) regulates prostaglandin metabolism. Conditional deletion of Hpgd in mouse Treg cells led to the buildup of functionally compromised Treg cells specifically in visceral adipose tissue, which in turn triggered local inflammation and systemic insulin resistance [31]. The identified NAD metabolism-related genes provide valuable insights into the pathogenesis of DN. Understanding these genes’ roles may lead to novel therapeutic targets, particularly in managing the distinct subtypes of DN. The differential expression of these genes suggests that interventions could be tailored to individual patients based on their NAD-related profiles, potentially improving treatment outcomes. The biomarkers identified in our study hold significant promise for clinical applications. Their correlation with renal function suggests they could serve as indicators for disease progression and treatment response. Incorporating these biomarkers into clinical practice may facilitate early diagnosis and personalized treatment plans for patients with DN.

Our findings provide mechanistic insights into how NAD metabolism influences DN. We specifically identified several key enriched pathways with distinct relevance to the pathogenesis of DN. Among these, the reactive oxygen species (ROS) pathway exhibited the higher enrichment in patients classified under subtype 1. This is particularly significant because the accumulation of ROS in diabetic kidneys contributes to podocyte apoptosis, mesangial cell proliferation, and the epithelial-mesenchymal transition of tubular cells [32, 33]. The enrichment of the fatty acid metabolism pathway in subtype 1 highlights the metabolic reprogramming occurring in diabetic kidneys, where increased lipid accumulation contributes to renal lipotoxicity and progressive fibrosis [34]. The enrichment of the glycolysis pathway in subtype 1 indicates a metabolic shift towards aerobic glycolysis in diabetic renal cells, which promotes inflammatory responses and extracellular matrix production [35]. The interferon alpha and gamma response pathways demonstrated significant enrichment in subtype 1, suggesting enhanced activation of the innate immune system. This contributes to chronic inflammation and progressive renal injury in DN [36]. Regarding immune cell infiltration, our analysis revealed specific patterns of immune dysregulation in DN subtypes. Subtype 1 demonstrated increased infiltration of macrophages, creating a pro-inflammatory microenvironment that perpetuates renal injury. This finding aligns with previous studies showing that macrophage accumulation in diabetic kidneys promotes inflammatory cytokine production and fibrosis progression [37]. Additionally, we observed increased T cell infiltration in subtype 1, which has been linked to direct cytotoxic effects on renal tubular cells and the promotion of interstitial fibrosis [38].

These pathways are crucial in understanding how dysregulation of NAD metabolism contributes to the development and progression of DN. Previous studies have shown that NAD+ depletion leads to increased oxidative stress and inflammation, which exacerbates renal injury in diabetic models [39–41]. This suggests that therapeutic strategies aimed at restoring NAD+ levels could potentially mitigate the adverse effects associated with DN. Furthermore, the interplay between NAD metabolism and mitochondrial function cannot be overlooked, as mitochondria are central to both energy production and apoptosis in renal cells [42, 43]. It is essential to explore how enhancing NAD+ synthesis might improve mitochondrial bioenergetics, thereby reducing oxidative damage and promoting cell survival. Additionally, the association of NAD with fatty acid metabolism highlights another critical avenue for research. The dysregulation of lipid metabolism in diabetes is well-documented, and it may be beneficial to investigate how NAD+ supplementation could recalibrate lipid profiles in renal tissues [44, 45]. By addressing the metabolic inflexibility often seen in diabetic patients, we might uncover novel preventive strategies against DN. Moreover, the differential responses of DN subtypes to interferon alpha and gamma indicate that immune modulation could play a role in disease progression [46, 47]. Future studies should focus on how NAD+ influences immune cell activation and function in the renal microenvironment. By delineating these mechanisms, we can potentially identify biomarkers for DN progression and therapeutic targets that could enhance renal resilience. Overall, the intricate relationship between NAD metabolism and various biological pathways suggests a multifaceted approach to understanding and treating DN. As we deepen our investigation into these connections, we may pave the way for innovative interventions that not only restore metabolic balance but also improve patient outcomes in diabetic kidney disease.

The strengths of our study include a comprehensive analysis of multiple datasets and the application of robust machine learning algorithms to identify key biomarkers. Despite these strengths, certain limitations exist, such as the reliance on existing datasets, which may not capture all aspects of NAD metabolism in DN. In this study, we utilized two datasets, GSE30528 and GSE30529, which include 19 DN samples and 25 control samples. While these datasets are relevant, they have limitations in terms of sample size and potential heterogeneity. The relatively small sample size may limit the statistical power and generalizability of our findings. Additionally, potential biases inherent in these datasets, such as differences in sample collection, processing, and population demographics, could affect the robustness of our results. Future studies should strive for validation in larger, more diverse cohorts.

Future research should also focus on clarifying the precise mechanisms by which NAD metabolism-related genes influence DN. Longitudinal studies could provide insight into the interactions of these genes with environmental and genetic factors over time. Additionally, exploring therapeutic interventions targeting NAD metabolism may yield promising management strategies for DN. For instance, clinical trials assessing the efficacy of NAD+ precursors in diabetic patients could offer valuable data on their potential benefits in preventing or treating DN.

In conclusion, our study underscores the significant role of NAD metabolism-related genes in DN. The identification of distinct subtypes and potential biomarkers paves the way for future research and therapeutic interventions, ultimately contributing to improved patient outcomes in DN management. By advancing our understanding of NAD metabolism, we can enhance the precision of diabetes care and foster a healthier society.

## Data Availability

The data utilized in this study were sourced from the GEO database and are publicly accessible at https://www.ncbi.nlm.nih.gov/geo/. The specific datasets analyzed include GSE30528, GSE30529, GSE96804, GSE104954, and GSE142025. We downloaded the raw data from the GEO website and conducted all bioinformatics analyses and visualizations using the processed data through the Xiantao Academic Online platform, which can be accessed at https://www.xiantaozi.com/. This platform also provides the relevant analysis scripts and code used in this study.

## Footnotes

1
https://nephroseq.org/
