# Supplementary material for: Role of NAD metabolism-related genes in diabetic nephropathy: subtype classification, biomarker identification, and association with renal function
Source: Exp Biol Med (Maywood). 2026 Jan 26;250:10601. doi: 10.3389/ebm.2025.10601 (PMC12883428; doi:10.3389/ebm.2025.10601)
Supplement: Supplementary file 2 [file Table1.docx]

Supplementary tables

Table S1 Information on the GEO dataset used for this study.

| GEO ID | Platform | Con group | DN group | Species | Tissue subregion | Application |
| --- | --- | --- | --- | --- | --- | --- |
| GSE30529 | GPL571 | 12 | 10 | Homo sapiens | Renal tubule | Analysis |
| GSE30528 | GPL571 | 13 | 9 | Homo sapiens | Glomerulus | Analysis |
| GSE96804 | GPL17586 | 20 | 41 | Homo sapiens | Glomerulus | Validation |
| GSE104954 | GPL22945 | 18 | 7 | Homo sapiens | Tubulointerstitium | Validation |
| GSE142025 | GPL20301 | 9 | 27 (advanced DN) | Homo sapiens | Glomerulus | Validation |

Supplementary figures


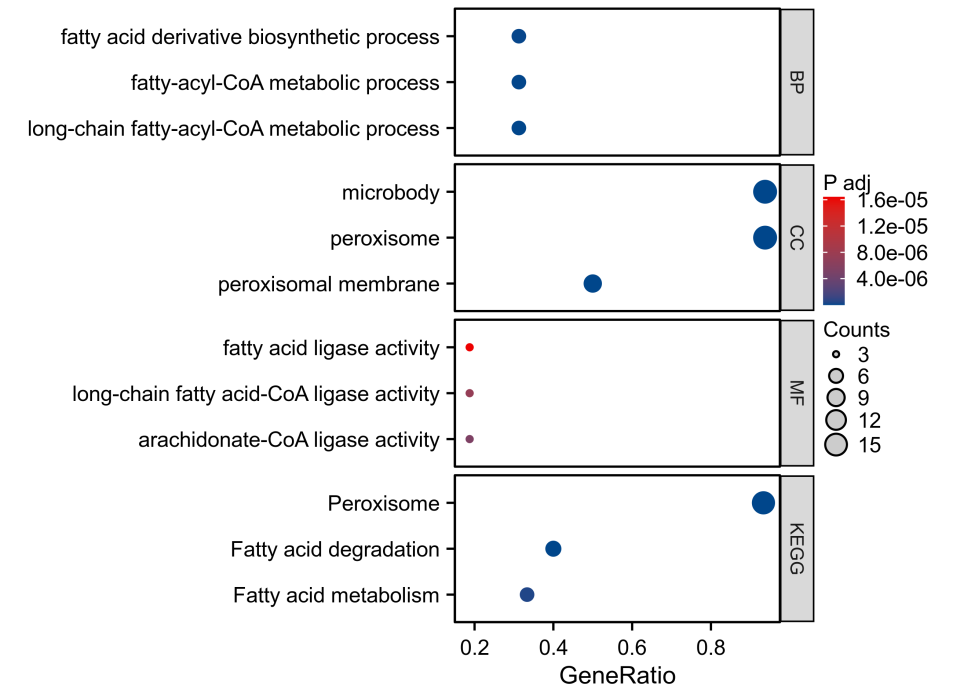


Figure S1 Enrichment analysis of DPGs. GeneRatio indicates the proportion of genes involved in each term, and dot size reflects the count of genes, with color representing the adjusted p-value (P adj).
